# The Impact of CB1 Receptor on Nuclear Receptors in Skeletal Muscle Cells

**DOI:** 10.3390/pathophysiology28040029

**Published:** 2021-10-05

**Authors:** Mansour Haddad

**Affiliations:** Department of Clinical Pharmacy, Faculty of Pharmacy, Philadelphia University, P.O. Box 1, Amman 19392, Jordan; dr.man.haddad@gmail.com

**Keywords:** cannabinoid CB1 receptors, ACEA, rimonabant, NR4A, skeletal muscle cells

## Abstract

Cannabinoids are abundant signaling compounds; their influence predominantly arises via engagement with the principal two G-protein-coupled cannabinoid receptors, CB1 and CB2. One suggested theory is that cannabinoids regulate a variety of physiological processes within the cells of skeletal muscle. Earlier publications have indicated that expression of CB1 receptor mRNA and protein has been recognized within myotubes and tissues of skeletal muscle from both murines and humans, thus representing a potentially significant pathway which plays a role in the control of skeletal muscular activities. The part played by CB1 receptor activation or inhibition with respect to these functions and relevant to targets in the periphery, especially skeletal muscle, is not fully delineated. Thus, the aim of the current research was to explore the influence of CB1 receptor stimulation and inhibition on downstream signaling of the nuclear receptor, NR4A, which regulates the immediate impacts of arachidonyl-2′-chloroethylamide (ACEA) and/or rimonabant in the cells of skeletal muscle. Murine L6 skeletal muscle cells were used in order to clarify additional possible molecular signaling pathways which contribute to alterations in the CB1 receptor. Skeletal muscle cells have often been used; it is well-documented that they express cannabinoid receptors. Quantitative real-time probe-based polymerase chain reaction (qRT-PCR) assays are deployed in order to assess the gene expression characteristics of CB1 receptor signaling. In the current work, it is demonstrated that skeletal muscle cells exhibit functional expression of CB1 receptors. This can be deduced from the qRT-PCR assays; triggering CB1 receptors amplifies both NR4A1 and NR4A3 mRNA gene expression. The impact of ACEA is inhibited by the selective CB1 receptor antagonist, rimonabant. The present research demonstrated that 10 nM of ACEA notably amplified mRNA gene expression of NR4A1 and NR4A3; this effect was suppressed by the addition of 100 nM rimonabant. Furthermore, the CB1 receptor antagonist led to the downregulation of mRNA gene expression of NR4A1, NR4A2 and NR4A3. In conclusion, in skeletal muscle, CB1 receptors were recognized to be important moderators of NR4A1 and NR4A3 mRNA gene expression; these actions may have possible clinical benefits. Thus, in skeletal muscle cells, a possible physiological expression of CB1 receptors was identified. It is as yet unknown whether these CB1 receptors contribute to pathways underlying skeletal muscle biological function and disease processes. Further research is required to fully delineate their role(s).

## 1. Introduction

Studies have shown that several physiological functions played by the majority of tissues are regulated by cannabinoids [1]. The same is the case for skeletal muscles [2,3]. It has been established that the endocannabinoid system (ECS) works as a complicated endogenous signaling system that is comprised of at least two cannabinoid receptors together with their endogenous ligands as well as enzymes that bring about biosynthesis and degradation of ligands [4]. Receptors of cannabinoids are members of the G-protein coupled receptors superfamily and they have been grouped as CB1 and CB2 receptors that participate in adenylate cyclase regulation [5]. A number of cells and tissues such as skeletal muscles express cannabinoid receptors [6,7,8]. The role of CB1 receptor as the main receptor bringing about the outcomes of the endocannabinoid system in metabolic processes. CB1 receptors have proven to be the most commonly expressed GPCRs in case of brain tissue [9], though they are also found in peripheral tissues like skeletal muscle, pancreas, liver and adipose tissue [10,11]. Conversely, CB2 receptors are mostly found to be expressed by constituents of immune system. In particular, they are expressed inside thymus, tonsils and spleen [12,13].

One of the largest tissues of human body is the skeletal muscle. In the case of rats, it constitutes majority of the body weight [14,15,16,17]. In general, it has been recognized as the main site of metabolism of fatty acids and glucose [18,19,20,21,22,23]. The role of skeletal muscle in sustaining body glucose homeostasis has proven to be very important [24]. Moreover, skeletal muscle serves as site of insulin resistance as well. As this tissue is capable of oxidizing fatty acid and glucose, its role in metabolic disorders is also important [25]. A number of scientists sometimes refer skeletal muscle as an endocrine organ considering discharge of inflammatory mediators by this tissue [17]. Taken together, skeletal muscle is essentially involved in glycemic control, sustaining glucose homeostasis and in regulating metabolic reactions of human system [24]. The skeletal muscle may perform the abovementioned functions through the endocannabinoid system; yet this possibility has not been investigated thoroughly.

NR4A is a subfamily of the orphan nuclear receptor superfamily consisting of three members, Nur77 (NR4A1), Nurr1 (NR4A2) and NOR1 (NR4A3) [26]. Several recent studies have demonstrated that NR4A receptors are key transcriptional regulators implicated in various biological processes, such as inflammation, lipid and glucose metabolism, insulin sensitivity, energy balance, and cell proliferation and differentiation [27,28]. These studies have focused on NR4A mainly in the liver, adipose, and skeletal muscle [29,30]. There is growing evidence to suggest that the activation of NR4A leads to an increase in the gene expression of intracellular downstream signaling pathways that potentially participate in the regulation of glucose and fatty acid metabolism and cell growth in skeletal muscle [31,32]. The NR4A family is also reported to play metabolic roles in all major insulin-sensitive target tissues. For example, NR4A1 is reported to be a factor in regulating glucose and lipid metabolism in muscle [33,34]. Cross-talk between the cannabinoid CB1 receptor and NR4A signaling may potentially represent an important yet unknown mechanism contributing to the regulation of skeletal muscle functions. Thus, further studies using L6 skeletal muscle cells as a cell model to understand the molecular signaling involved in the cross-talk are highly significant.

The CB1 receptor was found to be expressed in skeletal muscle [2], cannabinoid receptor ligands were shown to produce a CB1 receptor-dependent reduction in cAMP levels in transfected CHO cells [35] and different regions of the rat brain [36], and cAMP was found to be involved in an increased expression of NR4A in skeletal muscle [7,20,36,37]. Therefore, CB1 receptors can potentially affect the nuclear receptor subfamily 4, group A (NR4A), through the cAMP or Gβγ pathway in rat L6 skeletal muscle cell myotubes. Consequently, CB1 receptors may potentially modulate glucose and fatty acid metabolism and inflammation in skeletal muscle tissue. To address this issue, an investigation of the signaling events (NR4A family) underlying the activation and inhibition of the CB1 receptor in rat L6 skeletal muscle cells took place. The purpose of this work was to explain the potential signaling underlying the cannabinoid CB1 receptor modulation on NR4A mRNA gene expression.

## 2. Materials and Methods

Tocris Bioscience (Bristol, UK) supplied the insulin, arachidonyl-2′-chloroethylamide (ACEA), and rimonabant, while Santa Cruz (Dallas, TX, USA) provided the dimethylsulphoxide reagent. Thermo Scientific Company (Waltham, MA, USA) supplied the Maxima Probe qPCR Master Mix (2X) and Thermo Scientific RevertAid First Strand cDNA Synthesis, while Qiagen (Hilden, Germany) was the provider of the RNeasy Mini Total RNA Purification kits and RNase-Free DNase Set. Applied Biosystem (Waltham, MA, USA) provided Trizol and charcoal stripped serum, while FBS (fetal bovine serum) was obtained from Capricorn Scientific (Ebsdorfergrund, Germany). Sigma Company (Saint Louis, MO, USA) supplied horse serum, and Ham-F 10 was obtained from PAA Company (Cambridge, UK). Dulbecco’s modified essential medium (DMEM) was supplied by Caisson (Denver, CO, USA).

### 2.1. Cell Culture

**Figure 1:**
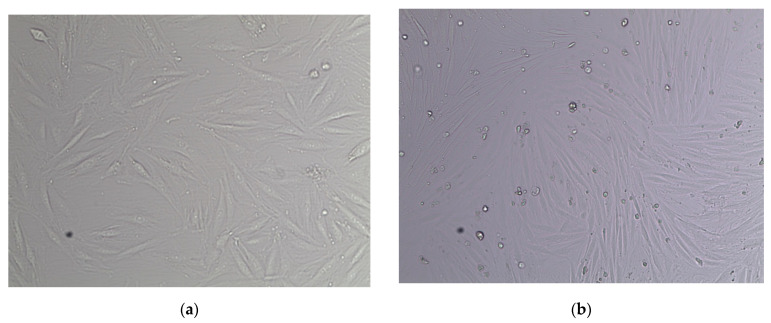

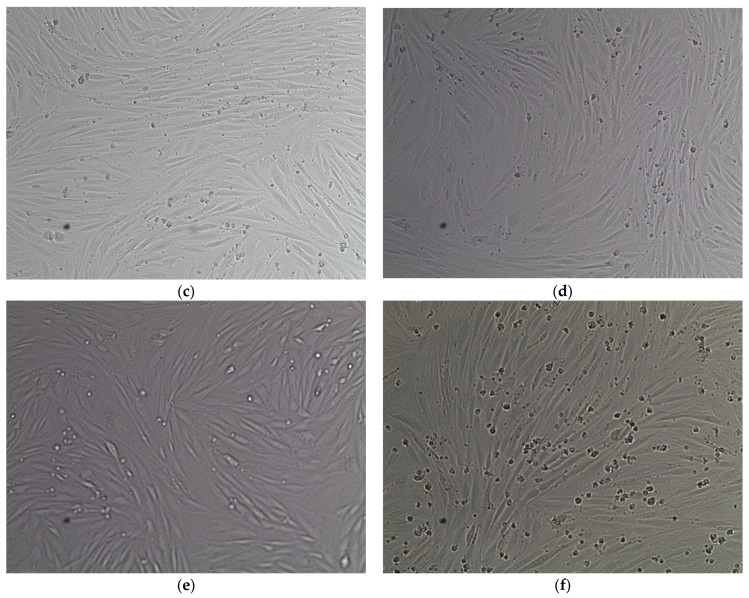
Images depicting L6 skeletal muscle myoblasts from rats, differentiated into L6 skeletal muscle myotubes (passage 7). (**a**) Myoblasts obtained on day 3 of the tissue culture in 10% FBS Ham F-10 media (10×). (**b**) Cells obtained on day 4 of the tissue culture in 6% horse serum Ham F-10 media (10×). (**c**) Myotubes obtained on day 5 of the tissue culture in 2% horse serum Ham F-10 media (10×). (**d**) Myotubes obtained after 4 h of tissue culture in 2% delipidated serum Ham F-10 media (10×). (**e**) Myotubes obtained after one hour of cell starvation in only Ham F-10 media (10×). (**f**) Myotubes obtained after 19 h of cell starvation in only Ham F-10 media (10×).

The American Type Culture Collection (Manassas, VA, USA) provided the L6 skeletal muscle cell line from rats along with the myoblast cell line, originating from cells which had been maintained in the form of an attached monolayer culture in DMEM which had a high glucose level (4500 mg/L) with L-glutamate which had been supplemented by 10% (*v*/*v*) heat-inactivated FBS and 100 µg/mL of penicillin-streptomycin. Incubation of the cells took place at a temperature of 37 °C using a 5% carbon dioxide atmosphere at 90% humidity. Passaging of the cells occurred at around 60–70% confluency, while changing of the medium was carried out thrice weekly, as shown in Figure 1. The confluent cells underwent 14 days of further culturing in 25 cm^2^ flasks in order to allow myotubes to form, in alignment with the protocols outlined in my previous publications [15,16,17], albeit with minor modifications (Figure 1). After around two weeks of culturing, the 70–90% confluent myotubes were then exposed to 2% (*v*/*v*) delipidated serum for a period of 5 h before undergoing starvation for a further 19 h. Figure 1 then shows that the cells were treated for varying periods of time (1, 3, 5, and 24 h) with vehicle (0.1% DMSO), rimonabant 100 nM, ACEA 10 nM, and insulin 100 nM. The ACEA and rimonabant cells had undergone pre-treatment using rimonabant for 10 min before adding ACEA. After the treatment, the cells were washed using ice-cold PBS, before lysing using Trizol (2 mL per flask).

### 2.2. Extraction of RNA and Synthesis of cDNA

The L6 skeletal muscle cells of rats were placed in 25 cm^2^ flasks and scraped in 2 mL of ice-cold Trizol, whereupon the RNA was separated and isolated in line with the guidelines of the manufacturer. RNeasy purification columns (Qiagen, Hilden, Germany) were then used to carry out the RNA clean-up and on-column DNase digestion. A spectrophotometer (JENWAY Genova Nano, Stafford, UK) was then used to assess the concentration and purity of the RNA. In order to carry out the cDNA synthesis, a quantity of 500 ng of total RNA underwent reverse transcription via RevertAid First Strand cDNA Synthesis in a process taking 5 min using a total volume of 20 µL at a temperature of 25 °C, before the temperature was increased to 42 °C for the subsequent one-hour period. Finally, termination of the reaction took place for 5 min at 70 °C. Gene expression was then quantified using the relative standard curve approach on the basis of the TaqMan quantitative real-time PCR (qRT-PCR). For this process, the preparation of the samples was carried out using a total reaction volume of 25 µL (comprising 13 µL Maxima Probe qPCR Master Mix 2× reagent, 1.5 µL of forward primer (10 µM), 2.5 µL Probe (2 µM), 1.5 µL of reverse primer (10 mM), 5 µL of water, and 5 µL of cDNA). A 7500 fast real-time PCR system (Applied Biosystems, Waltham, MA, USA) was employed to complete the qRT-PCR analysis, while the determination of the gene expression was made by considering the relationship to the reference gene, TATA. Primer Express software (Applied Biosystems, USA) was used in the case of probes and primers for all genes, as shown in Table 1, with the design and synthesis performed by Integrated DNA Technologies, Inc. (Coralville, IA, USA). The standard curve approach was employed, using a slope ranging from −3.2 to −3.6 with R^2^ values exceeding 99%, reflecting efficiency of amplification approaching 100%.

### 2.3. Data Analysis

Data are presented in the form of mean ± SEM following the generation of triplicate or quadruplicate wells from no fewer than three experimental groups. Data analysis of mRNA data employed one-way ANOVA and a Tukey test. The GraphPad Prism, version 5.03 (GraphPad Software Inc., San Diego, CA, USA) was used for all analyses, and the statistical significance level was determined to be *p* < 0.05.

## 3. Results

### 3.1. Effects of ACEA, Rimonabant, and Insulin on NR4R1 mRNA Gene Expression

**Figure 2:**
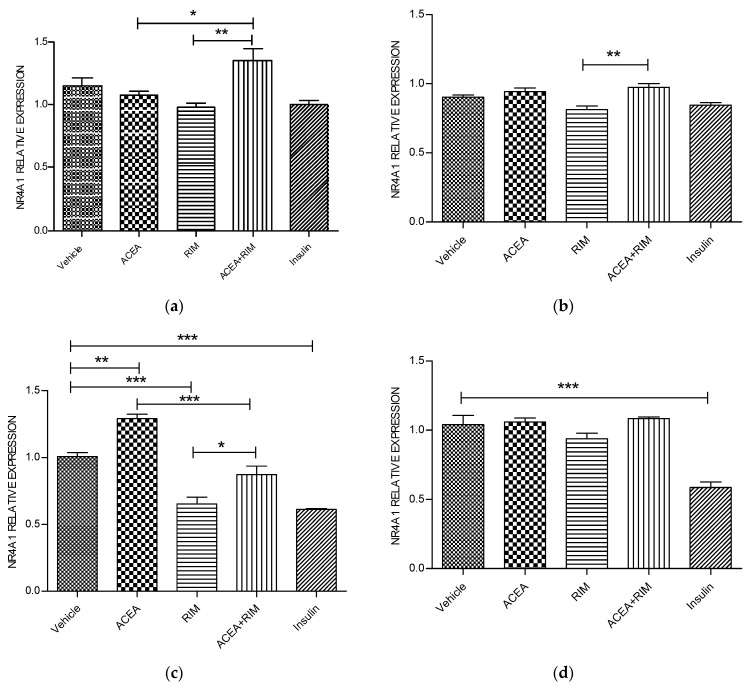
NR4A1 muscle myotubes—ACEA, rimonabant, ACEA and rimonabant, and insulin affect NR4A1 gene expression; myotubes fed with delipidated serum. The stimulation time covered 3 to 24 h, and NR4A1 mRNA levels, relative to TATA-Box, was evaluated by quantitative real-time PCR (qRT-PCR) (100 nM). The following scenarios explain the stimulation process conducted: (**a**) Stimulation was done for 1 h. (**b**) Stimulation was done for 3 h. (**c**) Stimulation was applied for up to 5 h. (**d**) Stimulation was applied for up to 24 h. The data were reported as the mean ± SEM of three separate groups. (n = 3; * denotes *p* < 0.05, ** denotes *p* < 0.01, and *** denotes *p* < 0.001). Data were investigated by conducting one-way ANOVA test and Tukey test. NR4A1; nuclear receptor subfamily 4, group A, member 1.

Using delipidated serum, treating the cells with ACEA (10 nM) for 5 h significantly up-regulated NR4A1 mRNA gene expression (*p* < 0.01). However, these responses were blocked by rimonabant (100 nM). The influence of ACEA on NR4A1 is therefore CB1 dependent. Interestingly, rimonabant significantly down-regulated NR4A1 mRNA gene expression (*p* < 0.001). Notably, using the delipidated serum, treating the cells with insulin (100 nM) for 5 or 24 h significantly down-regulated NR4A1 mRNA gene expression (*p* < 0.001) (Figure 2).

### 3.2. Effects of ACEA, Rimonabant, and Insulin on NR4R2 mRNA Gene Expression

**Figure 3:**
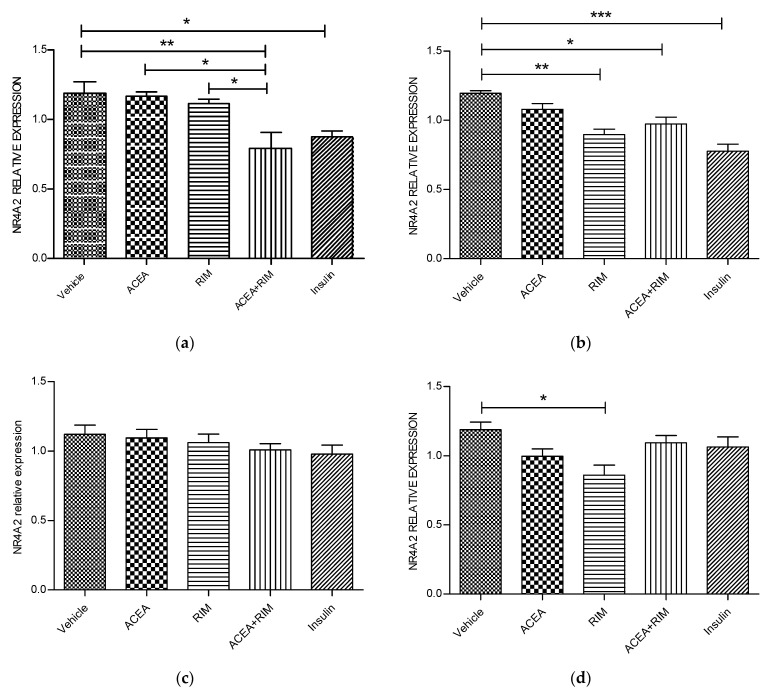
NR4A2 muscle myotubes—ACEA, rimonabant, ACEA and rimonabant, and insulin affect NR4A2 gene expression; myotubes fed with delipidated serum. The stimulation time covered 3 to 24 h, and NR4A2 mRNA levels, relative to TATA-Box, was evaluated by quantitative real-time PCR (qRT-PCR) (100 nM). The following scenarios explain the stimulation process conducted: (**a**) Stimulation was done for 1 h. (**b**) Stimulation was done for 3 h. (**c**) Stimulation was applied for up to 5 h. (**d**) Stimulation was applied for up to 24 h. The data were reported as the mean ± SEM of three separate groups. (n = 3; * denotes *p* < 0.05, ** denotes *p* < 0.01, and *** denotes *p* < 0.001). Data were investigated by conducting one-way ANOVA test and Tukey test. NR4A2; nuclear receptor subfamily 4, group A, member 2.

Using delipidated serum, treating the cells with rimonabant (100 nM) for 24 h significantly down-regulated NR4A2 gene expression (*p* < 0.05). By contrast, treating the cells with insulin for 1 or 3 h significantly down-regulated NR4A2 mRNA expression (Figure 3).

### 3.3. Effects of ACEA, Rimonabant and Insulin on NR4R3 mRNA Gene Expression

**Figure 4:**
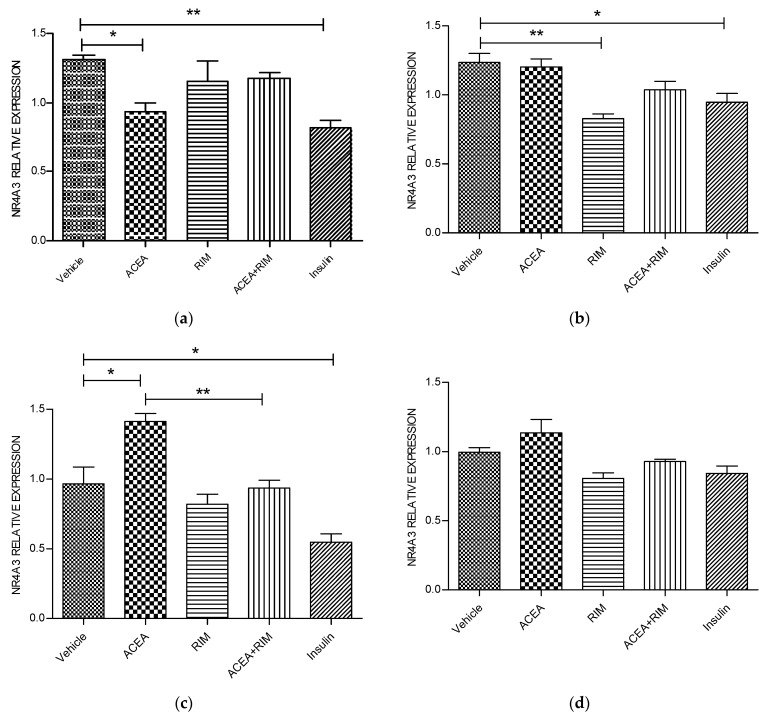
NR4A3 muscle myotubes—ACEA, rimonabant, ACEA and rimonabant, and insulin affect NR4A3 gene expression; myotubes fed with delipidated serum. The stimulation time covered 3 to 24 h, and NR4A3 mRNA levels, relative to TATA-Box, was evaluated by quantitative real-time PCR (qRT-PCR) (100 nM). The following scenarios explain the stimulation process conducted: (**a**) Stimulation was done for 1 h. (**b**) Stimulation was done for 3 h. (**c**) Stimulation was applied for up to 5 h. (**d**) Stimulation was applied for up to 24 h. The data were reported as the mean ± SEM of three separate groups. (n = 3; * denotes *p* < 0.05, and ** denotes *p* < 0.01). Data were investigated by conducting one-way ANOVA test and Tukey test. NR4A3; nuclear receptor subfamily 4, group A, member 1.

Using delipidated serum, treating the cells with ACEA (10 nM) for 5 h significantly up-regulated NR4A3 mRNA gene expression (*p* < 0.05). However, these responses were blocked by rimonabant (100 nM). The influence of ACEA on NR4A3 is therefore CB1 dependent. Interestingly, treating the cells with rimonabant (100 nM) for 1 h and 3 h significantly down-regulated NR4A3 gene expression (*p* < 0.05 and *p* < 0.05, respectively). Notably, using the delipidated serum, treating the cells with insulin (100 nM) for 1, 3 or 5 h significantly down-regulated NR4A3 mRNA gene expression (Figure 4).

## 4. Discussion

According to the novel findings of the present study, endocannabinoid analogue ACEA raises the gene expression of mRNA related to NR4A1 and NR4A3 in skeletal muscles. It has an effect through ACEA facilitated by the subtype cannabinoid CB1 receptor. To our knowledge, it is the first research about the effect of ACEA and its cannabinoid CB1 receptor subtypes on nuclear receptors (NR4A) in skeletal muscles, and it characterizes a novel mechanism of signaling for the cannabinoid CB1 receptor’s role in the cells of skeletal muscles. In the current research, the functionality of CB1 receptor was examined by evaluating the effect of CB1 receptor agonism (ACEA) or antagonism (rimonabant) on the activation of significant genes (nuclear receptors; NR4A1, NR4A2 and NR4A3) that might be involved in mitogenic, inflammatory and metabolic functions in rat skeletal muscle cells. Therefore, this study might also represent evidence that the CB1 receptor is potentially functionally active regarding inflammation and possibly in mitogenic and metabolic functions and for glucose and fatty acid metabolism.

Interestingly, in the current study, insulin 10 nM downregulated NR4A1, NR4A2 and NR4A3 expression in skeletal muscle cells using these time frame. Those NR4A expression was similarly inhibited by rimonabant. Rimonabant, a selective cannabinoid CB1 receptor antagonist/inverse agonist, was proven to inhibit the gene expression engaged in glycolysis proteins, glucose oxidation, insulin resistance and metabolism, transportation of fatty acids and their oxidation and regulation of energy and its metabolism, proliferation, differentiation and myogenesis (NR4A1, NR4A2 and NR4A3) in this study. Those NR4A expression was likewise reduced by insulin. In terms of the effect of ACEA, the expressions of genes (NR4A1 and NR4A3) have been substantially contrasted to those of insulin and rimonabant. The current pharmacological investigation makes use of rimonabant, a selective cannabinoid CB1 receptor antagonist, and ACEA, a specific cannabinoid CB1 receptor agonist. As a result, rimonabant’s impact provides convincing evidence for the participation of cannabinoid CB1 receptors at the very least. The selective cannabinoid receptor antagonist rimonabant was shown to enhance the gene expression involved in insulin sensitivity, glucose uptake, myogenesis and other metabolic processes which is in line with the same effect of insulin for those genes. This agrees with previous literature on skeletal muscle [38,39,40] that found the activation of CB1R in skeletal muscle cells is associated with insulin resistance, and impaired metabolic function, owing to increased energy intake and storage, impaired glucose and lipid utilization, and enhanced oxidative stress. This conclusion does not conflict with a previous researcher [27], who found that—in adipose cells (3T3-L1 cells), but not in skeletal muscle—the activation of NR4A receptors is known to promote glucose utilization by enhancing the activity of insulin to stimulate glucose transport since this research study used a different cell line culture.

ACEA was also employed, as it is 2000-times more specific for CB1 receptors than CB2 receptors [35]. In this current work, ACEA was observed to boost NR4A1 and NR4A3 mRNA expression in rat skeletal muscle myotubes. Rimonabant, a selective CB1 receptor antagonist/inverse agonist, was shown to prevent this ACEA-induced action. Because ACEA is a selective CB1 receptor agonist at the dose utilized in this current study, this data implies that NR4A1 and NR4A3 activation was mediated through CB1 receptor activation. In skeletal muscles, the CB1 receptor is an active receptor based on functions, according to this research. The following are the outcomes of our research. (I) The selective cannabinoid CB1 receptor agonist ACEA boosts NR4A1 and NR4A3 expression. The purpose of testing the action of this agonist at a concentration of 10 nM [35] was to test this hypothesis. (II) Rimonabant, a selective antagonist of the cannabinoid CB1 receptor subtype and CB1 inverse agonists and antagonists [38,41], blocked these responses. These data, taken together, offer significant evidence for the participation of cannabinoid CB1 receptors in ACEA-induced upregulation of NR4A expression. (III) Rimonabant suppresses the expression of the NR4A mRNA gene.

In this current study, the activation of cannabinoid CB1 receptor has been found to enhance expression of NR4A in skeletal muscles. So, CB1 receptors can moderate metabolism of glucose and fat in skeletal muscles. It is reinforced by the circumstance that (1) NR4A was found to be decreased in the skeletal muscles among diabetic animals [27], (2) NR4A is linked with genes linked with fatty acid and glucose utilization via mRNA up-regulated expression of FOXO1, PDK4, lipin-1α, and PGC-1α [42], (3) after feeding the high-fat diet, NR4A null mice was compared with wild-type animals that presented reduced mRNA expression of PDK4, Lipin 1α, and GLUT4 along with impaired insulin resistance and phosphorylation of insulin receptor substrate 1 (IRS-1) in skeletal muscles and reduced clearance of blood glucose, higher body weight and low energy consumption [28], (4) C2C12 siRNA-NR4A cells were presented to reduce the mRNA expression in C2C12 cells of fatty acid translocase (CD36/fat), uncoupling GLUT4 and protein3 (UCP3) than that of wild-type native C2C12 cells [43,44] and (5) non-insulin glucose consumption was revealed to raise significantly NR4A expression mediated by adenovirus in C2C12 cells than those of normal C2C12 cells [45,46]. Based on this, the CB1 receptor modulation through ligands may affect utilization of fatty acid and glucose in skeletal muscles. As a result, CB1 receptor agonists/antagonists could be explored as a potential therapeutic option in patients with diabetes or adiposity. Additional research will be conducted in the future to elucidate this point.

NR4A activation has been linked to enhanced gene expression of various metabolic genes in a variety of tissues [47], particularly in skeletal muscles. Activation of NR4A has been linked to muscle growth and development, glucose metabolism, and oxidation of fatty acid [34,43]. As a result, it is probable that ACEA exerts at least the effects listed earlier in skeletal muscle, and that these actions are facilitated via activation of cannabinoid CB1 receptors. Cannabinoid receptors, namely cannabinoid CB1 receptors, have been shown to regulate a variety of cellular responses implicated in obesity and glucose homeostasis formerly [1,39,40]. Because cannabinoid CB1 receptors increased NR4A mRNA gene expression in skeletal muscles and NR4A1 restrains inflammation, glucose transport, and insulin action [45,48], it is possible that cannabinoid CB1 receptors modulate skeletal muscle physiological roles including glucose and fatty acid metabolism. The function of these genes in muscle tissue are little understood in the research.

Using rimonabant, a unique therapeutic intervention in the treatment of hyperglycemia and obesity might occur through the antagonism of the endocannabinoid system. In fact, studies from animals and humans showed an increase in the levels of endocannabinoids in the obese state. In addition, obese animal models showed that the levels of endocannabinoids were increased in the peripheral and hypothalamus tissues [49,50,51]. Furthermore, previous studies demonstrated that circulating levels of endocannabinoids including anandamide (AEA) and 2-Arachidonoylglycerol (2-AG) were raised in visceral adipose tissue in hyperglycaemic type 2 diabetic and obese patients [49,52,53]. Moreover, CB_1_ knock-out mice were found to be resistant to diet-induced obesity [50,54]. Originally, CB_1_ receptor antagonism was also realized to potentially enhance metabolic parameters [55,56,57].

CB1 receptor expression has been reported in rodent and human skeletal muscle in past studies [2]. CB1 receptor protein expression was observed to be considerably lower in obese Zucker rats’ soleus muscle compared to lean Zucker rats [40]. CB1 receptor mRNA expression in the soleus muscle of C56BL/6 mice was likewise observed to be higher following high fat eating [1,49]. The G proteins of the Gi/o family are involved in CB1 receptor signaling [58]. Pertussis toxin, in fact, decreased the effect of CB1 receptor activation [59]. As a result, Gi/o inhibits adenylyl cyclase and, as a result, cAMP buildup. Ion channels can also be regulated by Gi/o, with calcium channels being inhibited [60,61,62] and activating potassium channels through Gβγ [63,64,65]. Further studies are required to be performed to examine the mechanism beyond the NR4A signaling in response to CB1 receptor activation/inhibition.

Rimonabant may also act as an agonist through other receptors like GPR55, according to some research [66]. It is also worth noting that rimonabant can also work as an inverse agonist. So, the explanation of rimonabant response is very difficult to describe. On the basis of current data, it is also impossible to say if the effects of rimonabant are depending on CB1 receptor inhibition (a CB1 receptor dependent manner). More studies, such as employing charcoal stripped serum, must be done to get a precise, comprehensive picture of these concerns. To better understand these effects, more research is needed, for example utilizing GPR55 antagonist or CB1 receptor siRNA. Of note, it can be suggested that there is a need for more studies assessing protein expression level. Additionally, more work is also suggested to assess the direct effect of cannabinoid CB1 receptor on NR4A using knock out of CB1 receptor in L6 skeletal muscle cells or to mimic the muscle physiology using knock out of CB1 receptor in primary myoblast isolated from mice or rats. This could provide more evidence with regards to this signaling. Other areas that could benefit from more studies include assessing more important genes connected to this signaling in skeletal muscle such as PDK4 and CPT1B and assessing functional assays such as glucose uptake and fatty acid oxidation.

## 5. Conclusions

On the basis of these outcomes, it has been established that ACEA raises the mRNA expression of NR4A1 and NR4A3 frequently through the CB1 cannabinoid receptor signaling pathway. In addition, the expression of NR4A mRNA was found to be down-regulated by rimonabant. In the skeletal muscles, the cannabinoid CB1 receptors are expressed efficiently and often signals via NR4A pathways. Cannabinoid CB1 receptor antagonists/agonists/inverse agonists can be a useful mediator to assist the skeletal muscles in functional activities. In fact, the cannabinoid CB1 receptors are novel and highly significant drug targets in the curing and therapeutic management of inflammatory and metabolic diseases. Overall, it was found that the CB1 receptor is significantly efficient in skeletal muscles of rats. More research is necessary to validate the detailed part of endocannabinoids in the gene expression regulation in skeletal muscles and its significance in the development of glucose and fatty acid metabolism, inflammation, proliferation, myogenesis, obesity, and insulin resistance. This might also support evidence for possible potential roles of the CB1 receptor in skeletal muscle, and this may have general implications for diabetes mellitus, obesity, inflammation and wound healing.

## 6. Significance

This study describes a previously undiscovered signalling system in skeletal muscle cells that involves cannabinoid CB1 receptors. Furthermore, it explains a previously unknown process of skeletal muscle nuclear signalling to reveal another possible therapeutic target for at least metabolic diseases.

## Figures and Tables

**Table 1:** List of gene primer and probe sequences.

Gene	Sequences (5′ → 3′)	Amplicon Size (bp)
NR4A1	Probe 5′-CTTTATCCTCCGCCTGGCCTACCGA-3′ Forward primer 5′-TGTTGCTAGAGTCCGCCTTTC-3′ Reverse primer 5′-CAGGCCTGAGCAGAAGATGAG-3′	95
NR4A2	Probe 5′-TACGCTTAGCATACAGGTCCAACCCAGTG-3′ Forward Primer 5′-CCAAAGCCGATCAGGACCT-3′ Reverse primer 5′-GACCACCCCATTGCAAAAGAT-3′	116
NR4A3	Probe: 5′-ACTGTCCCACCGACCAGGCCACT-3′ Forward Primer: 5′-GACGCAACGCCCAGAGAC-3′ Reverse primer 5′-TAGAACTGCTGCACGTGCTCA-3′	92
TATA-BOX	Probe 5′-TCCCAAGCGGTTTGCTGCAGTCA-3′ Forward Primer 5′-TTCGTGCCAGAAATGCTGAA-3′ Reverse Primer 5′-GTTCGTGGCTCTCTTATTCTCATG-3	73

## Data Availability

The data used to support the findings of this study are available from the corresponding author upon request.

## References

[1] Pagotto U., Marsicano G., Cota D., Lutz B., Pasquali R. (2006). The emerging role of the endocannabinoid system in endocrine regulation and energy balance. Endocr. Rev..

[2] Cavuoto P., McAinch A.J., Hatzinikolas G., Janovska A., Game P., Wittert G.A. (2007). The expression of receptors for endocannabinoids in human and rodent skeletal muscle. Biochem. Biophys. Res. Commun..

[3] Bielawiec P., Harasim-Symbor E., Konstantynowicz-Nowicka K., Sztolsztener K., Chabowski A. (2020). Chronic cannabidiol administration attenuates skeletal muscle de novo ceramide synthesis pathway and related metabolic effects in a rat model of high-fat diet-induced obesity. Biomolecules.

[4] Ge D., Odierna G.L., Phillips W.D. (2020). Influence of cannabinoids upon nerve-evoked skeletal muscle contraction. Neurosci. Lett..

[5] Simcocks A.C., O’Keefe L., Jenkin K.A., Cornall L.M., Grinfeld E., Mathai M.L., Hryciw D.H., McAinch A.J. (2020). The Role of Atypical Cannabinoid Ligands O-1602 and O-1918 on Skeletal Muscle Homeostasis with a Focus on Obesity. Int. J. Mol. Sci..

[6] Haddad M. (2013). Do CB1 Cannabinoid Receptors Regulate Insulin Signalling in Rat Primary Skeletal Muscle Cells?. J. Phys. Pharm. Adv..

[7] Haddad M. (2014). What Does Rimonabant Do in Rat Primary Skeletal Muscle Cells?. Bio. Pharm. J..

[8] Haddad M. (2014). mRNA expression of GPCRs in rat skeletal muscle tissues. Int. J. Biol. Pharm. Allied Sci..

[9] Tsou K., Brown S., Sanudo-Pena M.C., Mackie K., Walker J.M. (1998). Immunohistochemical distribution of cannabinoid CB1 receptors in the rat central nervous system. Neuroscience.

[10] Izzo A.A., Sharkey K.A. (2010). Cannabinoids and the gut: New developments and emerging concepts. Pharmacol. Ther..

[11] Starowicz K.M., Cristino L., Matias I., Capasso R., Racioppi A., Izzo A.A., Di Marzo V. (2008). Endocannabinoid dysregulation in the pancreas and adipose tissue of mice fed with a high-fat diet. Obesity.

[12] Liu Q.R., Pan C.H., Hishimoto A., Li C.Y., Xi Z.X., Llorente-Berzal A., Viveros M.P., Ishiguro H., Arinami T., Onaivi E.S. (2009). Species differences in cannabinoid receptor 2 (CNR2 gene): Identification of novel human and rodent CB2 isoforms, differential tissue expression and regulation by cannabinoid receptor ligands. Genes Brain Behav..

[13] Brown S.M., Wager-Miller J., Mackie K. (2002). Cloning and molecular characterization of the rat CB2 cannabinoid receptor. Biochim. Biophys. Acta.

[14] Zurlo F., Larson K., Bogardus C., Ravussin E. (1990). Skeletal muscle metabolism is a major determinant of resting energy expenditure. J. Clin. Investig..

[15] Haddad M. (2021). Impact of Adenosine A2 Receptor Ligands on BCL2 Expression in Skeletal Muscle Cells. Appl. Sci..

[16] Haddad M. (2021). Impact of Adenosine Analogue, Adenosine-5’-N-Ethyluronamide (NECA), on Insulin Signaling in Skeletal Muscle Cells. Biomed. Res. Int..

[17] Haddad M. (2021). The Impact of CB1 Receptor on Inflammation in Skeletal Muscle Cells. J. Inflamm Res..

[18] Haddad M. (2014). Adenosine Receptors Machinery and Purinergic Receptors in Rat Primary Skeletal Muscle Cells. Biomed. Bio. Pharm. J..

[19] Haddad M. (2017). Adenosine A2B Receptors—Mediated Induction of Interleukin-6 in Skeletal Muscle Cells. Turk. J. Pharm. Sci..

[20] Haddad M. (2016). The Impact Of Adenosine A2B Receptors Modulation On Nuclear Receptors (NR4A) Gene Expression. Bio. Pharm. J..

[21] Haddad M. (2016). The impact of adenosine A2B receptors on glycolysis and insulin resistance in skeletal muscle. Int. J. Pharm. Sci. Res..

[22] Haddad M. (2016). The impact of adenosine A2B receptors modulation on peroxisome proliferator-activated receptor gamma co-activator 1-alpha and transcription factors. Int. J. Pharm. Sci. Res..

[23] Haddad M. (2016). The effect of NECA, CGS21680, PSB603 on fatty acid transport and oxidation in skeletal muscle cells. Int. J. Pharm. Sci. Res..

[24] Toft I., Bonaa K.H., Jenssen T. (1998). Insulin resistance in hypertension is associated with body fat rather than blood pressure. Hypertension.

[25] Cahova M., Vavrinkova H., Kazdova L. (2007). Glucose-fatty acid interaction in skeletal muscle and adipose tissue in insulin resistance. Physiol Res..

[26] Ranhotra H.S. (2015). The NR4A orphan nuclear receptors: Mediators in metabolism and diseases. J. Recept Signal. Transduct Res..

[27] Fu Y., Luo L., Luo N., Zhu X., Garvey W.T. (2007). NR4A orphan nuclear receptors modulate insulin action and the glucose transport system: Potential role in insulin resistance. J. Biol. Chem..

[28] Chao L.C., Zhang Z., Pei L., Saito T., Tontonoz P., Pilch P.F. (2007). Nur77 coordinately regulates expression of genes linked to glucose metabolism in skeletal muscle. Mol. Endocrinol..

[29] Kawasaki E., Hokari F., Sasaki M., Sakai A., Koshinaka K., Kawanaka K. (2009). Role of local muscle contractile activity in the exercise-induced increase in NR4A receptor mRNA expression. J. Appl. Physiol..

[30] Oita R.C., Mazzatti D.J., Lim F.L., Powell J.R., Merry B.J. (2009). Whole-genome microarray analysis identifies up-regulation of Nr4a nuclear receptors in muscle and liver from diet-restricted rats. Mech. Ageing Dev..

[31] Tontonoz P., Cortez-Toledo O., Wroblewski K., Hong C., Lim L., Carranza R., Conneely O., Metzger D., Chao L.C. (2015). The orphan nuclear receptor Nur77 is a determinant of myofiber size and muscle mass in mice. Mol. Cell Biol..

[32] Zhang W. (2014). MINOR (NR4A3) Overexpression in Mouse Skeletal Muscle Enhances Insulin Action. Mol. Genet. Med..

[33] Safe S., Jin U.H., Morpurgo B., Abudayyeh A., Singh M., Tjalkens R.B. (2015). Nuclear receptor 4A (NR4A) family—Orphans no more. J. Steroid. Biochem. Mol. Biol..

[34] Close A.F., Rouillard C., Buteau J. (2013). NR4A orphan nuclear receptors in glucose homeostasis: A minireview. Diabetes Metab..

[35] Hillard C.J., Manna S., Greenberg M.J., DiCamelli R., Ross R.A., Stevenson L.A., Murphy V., Pertwee R.G., Campbell W.B. (1999). Synthesis and characterization of potent and selective agonists of the neuronal cannabinoid receptor (CB1). J. Pharmacol. Exp. Ther..

[36] Bidaut-Russell M., Devane W.A., Howlett A.C. (1990). Cannabinoid receptors and modulation of cyclic AMP accumulation in the rat brain. J. Neurochem..

[37] Pearen M.A., Ryall J.G., Maxwell M.A., Ohkura N., Lynch G.S., Muscat G.E. (2006). The orphan nuclear receptor, NOR-1, is a target of beta-adrenergic signaling in skeletal muscle. Endocrinology.

[38] Esposito I., Proto M.C., Gazzerro P., Laezza C., Miele C., Alberobello A.T., D’Esposito V., Beguinot F., Formisano P., Bifulco M. (2008). The cannabinoid CB1 receptor antagonist rimonabant stimulates 2-deoxyglucose uptake in skeletal muscle cells by regulating the expression of phosphatidylinositol-3-kinase. Mol. Pharmacol..

[39] Pagotto U., Pasquali R. (2006). Endocannabinoids and energy metabolism. J. Endocrinol Investig..

[40] Lindborg K.A., Jacob S., Henriksen E.J. (2011). Effects of Chronic Antagonism of Endocannabinoid-1 Receptors on Glucose Tolerance and Insulin Action in Skeletal Muscles of Lean and Obese Zucker Rats. Cardiorenal Med..

[41] Carai M.A., Colombo G., Gessa G.L. (2005). Rimonabant: The first therapeutically relevant cannabinoid antagonist. Life Sci..

[42] Pearen M.A., Myers S.A., Raichur S., Ryall J.G., Lynch G.S., Muscat G.E. (2008). The orphan nuclear receptor, NOR-1, a target of beta-adrenergic signaling, regulates gene expression that controls oxidative metabolism in skeletal muscle. Endocrinology.

[43] Maxwell M.A., Muscat G.E. (2006). The NR4A subgroup: Immediate early response genes with pleiotropic physiological roles. Nucl. Recept Signal..

[44] Maxwell M.A., Cleasby M.E., Harding A., Stark A., Cooney G.J., Muscat G.E. (2005). Nur77 regulates lipolysis in skeletal muscle cells. Evidence for cross-talk between the beta-adrenergic and an orphan nuclear hormone receptor pathway. J. Biol. Chem..

[45] Chao L.C., Wroblewski K., Zhang Z., Pei L., Vergnes L., Ilkayeva O.R., Ding S.Y., Reue K., Watt M.J., Newgard C.B. (2009). Insulin resistance and altered systemic glucose metabolism in mice lacking Nur77. Diabetes.

[46] Chao L.C., Bensinger S.J., Villanueva C.J., Wroblewski K., Tontonoz P. (2008). Inhibition of adipocyte differentiation by Nur77, Nurr1, and Nor1. Mol. Endocrinol..

[47] Huang Q., Xue J., Zou R., Cai L., Chen J., Sun L., Dai Z., Yang F., Xu Y. (2014). NR4A1 is associated with chronic low-grade inflammation in patients with type 2 diabetes. Exp. Ther. Med..

[48] Eckardt K., May C., Koenen M., Eckel J. (2007). IGF-1 receptor signalling determines the mitogenic potency of insulin analogues in human smooth muscle cells and fibroblasts. Diabetologia.

[49] Matias I., Gonthier M.P., Orlando P., Martiadis V., De Petrocellis L., Cervino C., Petrosino S., Hoareau L., Festy F., Pasquali R. (2006). Regulation, function, and dysregulation of endocannabinoids in models of adipose and beta-pancreatic cells and in obesity and hyperglycemia. J. Clin. Endocrinol. Metab..

[50] Osei-Hyiaman D., DePetrillo M., Pacher P., Liu J., Radaeva S., Batkai S., Harvey-White J., Mackie K., Offertaler L., Wang L. (2005). Endocannabinoid activation at hepatic CB1 receptors stimulates fatty acid synthesis and contributes to diet-induced obesity. J. Clin. Investig..

[51] Di Marzo V., Goparaju S.K., Wang L., Liu J., Batkai S., Jarai Z., Fezza F., Miura G.I., Palmiter R.D., Sugiura T. (2001). Leptin-regulated endocannabinoids are involved in maintaining food intake. Nature.

[52] Engeli S., Bohnke J., Feldpausch M., Gorzelniak K., Janke J., Batkai S., Pacher P., Harvey-White J., Luft F.C., Sharma A.M. (2005). Activation of the peripheral endocannabinoid system in human obesity. Diabetes.

[53] Bluher M., Engeli S., Kloting N., Berndt J., Fasshauer M., Batkai S., Pacher P., Schon M.R., Jordan J., Stumvoll M. (2006). Dysregulation of the peripheral and adipose tissue endocannabinoid system in human abdominal obesity. Diabetes.

[54] Ravinet Trillou C., Delgorge C., Menet C., Arnone M., Soubrie P. (2004). CB1 cannabinoid receptor knockout in mice leads to leanness, resistance to diet-induced obesity and enhanced leptin sensitivity. Int. J. Obes. Relat. Metab. Disord..

[55] Liu Y.L., Connoley I.P., Wilson C.A., Stock M.J. (2005). Effects of the cannabinoid CB1 receptor antagonist SR141716 on oxygen consumption and soleus muscle glucose uptake in Lep(ob)/Lep(ob) mice. Int. J. Obes..

[56] Bermudez-Siva F.J., Serrano A., Diaz-Molina F.J., Sanchez Vera I., Juan-Pico P., Nadal A., Fuentes E., Rodriguez de Fonseca F. (2006). Activation of cannabinoid CB1 receptors induces glucose intolerance in rats. Eur. J. Pharmacol..

[57] Nogueiras R., Veyrat-Durebex C., Suchanek P.M., Klein M., Tschop J., Caldwell C., Woods S.C., Wittmann G., Watanabe M., Liposits Z. (2008). Peripheral, but not central, CB1 antagonism provides food intake-independent metabolic benefits in diet-induced obese rats. Diabetes.

[58] Munro S., Thomas K.L., Abu-Shaar M. (1993). Molecular characterization of a peripheral receptor for cannabinoids. Nature.

[59] Felder C.C., Joyce K.E., Briley E.M., Glass M., Mackie K.P., Fahey K.J., Cullinan G.J., Hunden D.C., Johnson D.W., Chaney M.O. (1998). LY320135, a novel cannabinoid CB1 receptor antagonist, unmasks coupling of the CB1 receptor to stimulation of cAMP accumulation. J. Pharmacol. Exp. Ther.

[60] Caulfield M.P., Brown D.A. (1992). Cannabinoid receptor agonists inhibit Ca current in NG108-15 neuroblastoma cells via a pertussis toxin-sensitive mechanism. Br. J. Pharmacol..

[61] Gebremedhin D., Lange A.R., Campbell W.B., Hillard C.J., Harder D.R. (1999). Cannabinoid CB1 receptor of cat cerebral arterial muscle functions to inhibit L-type Ca2+ channel current. Am. J. Physiol..

[62] Mackie K., Devane W.A., Hille B. (1993). Anandamide, an endogenous cannabinoid, inhibits calcium currents as a partial agonist in N18 neuroblastoma cells. Mol. Pharmacol..

[63] Turu G., Hunyady L. (2009). Signal transduction of the CB1 cannabinoid receptor. J. Mol. Endocrinol..

[64] Mackie K., Lai Y., Westenbroek R., Mitchell R. (1995). Cannabinoids activate an inwardly rectifying potassium conductance and inhibit Q-type calcium currents in AtT20 cells transfected with rat brain cannabinoid receptor. J. Neurosci..

[65] McAllister S.D., Griffin G., Satin L.S., Abood M.E. (1999). Cannabinoid receptors can activate and inhibit G protein-coupled inwardly rectifying potassium channels in a xenopus oocyte expression system. J. Pharmacol. Exp. Ther.

[66] Godlewski G., Offertaler L., Wagner J.A., Kunos G. (2009). Receptors for acylethanolamides-GPR55 and GPR119. Prostaglandins Lipid. Mediat..

